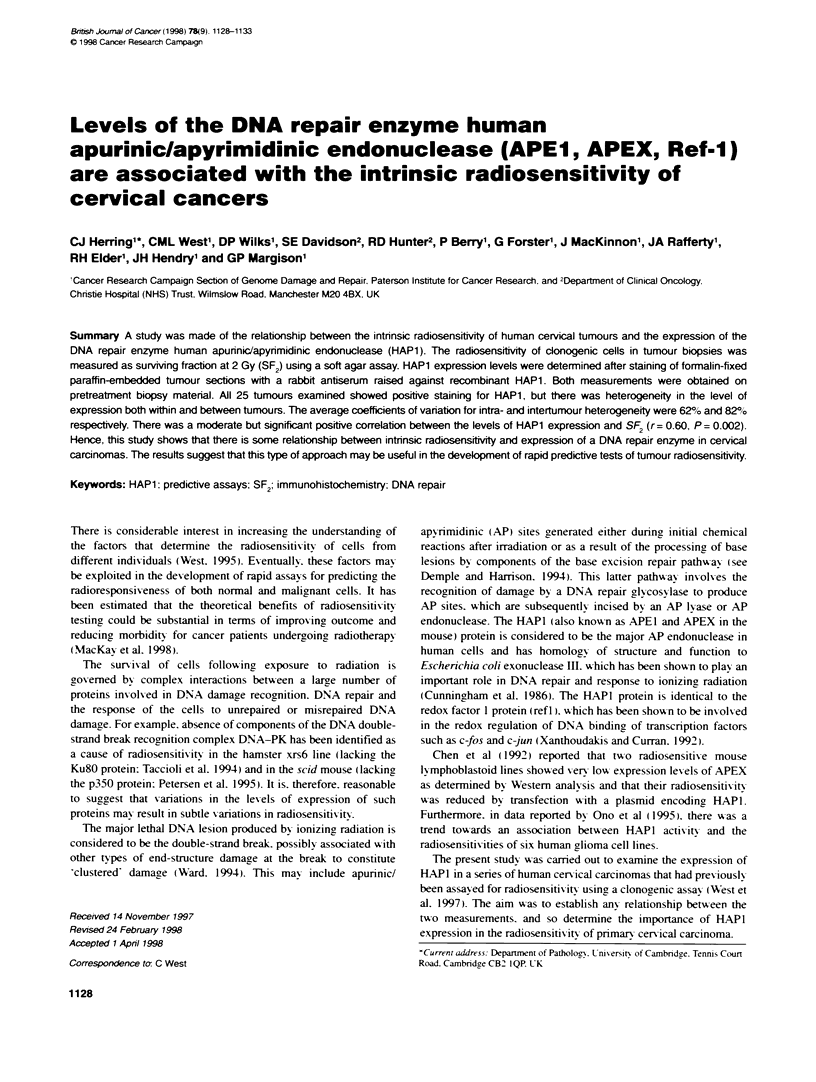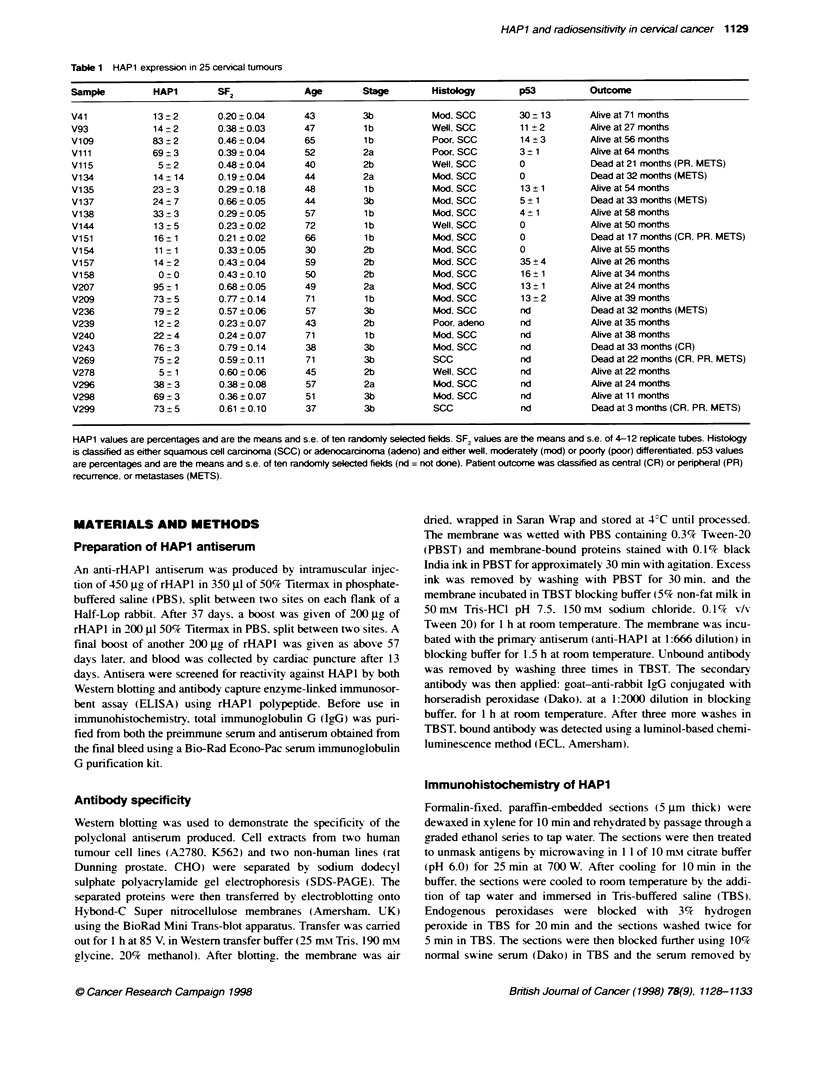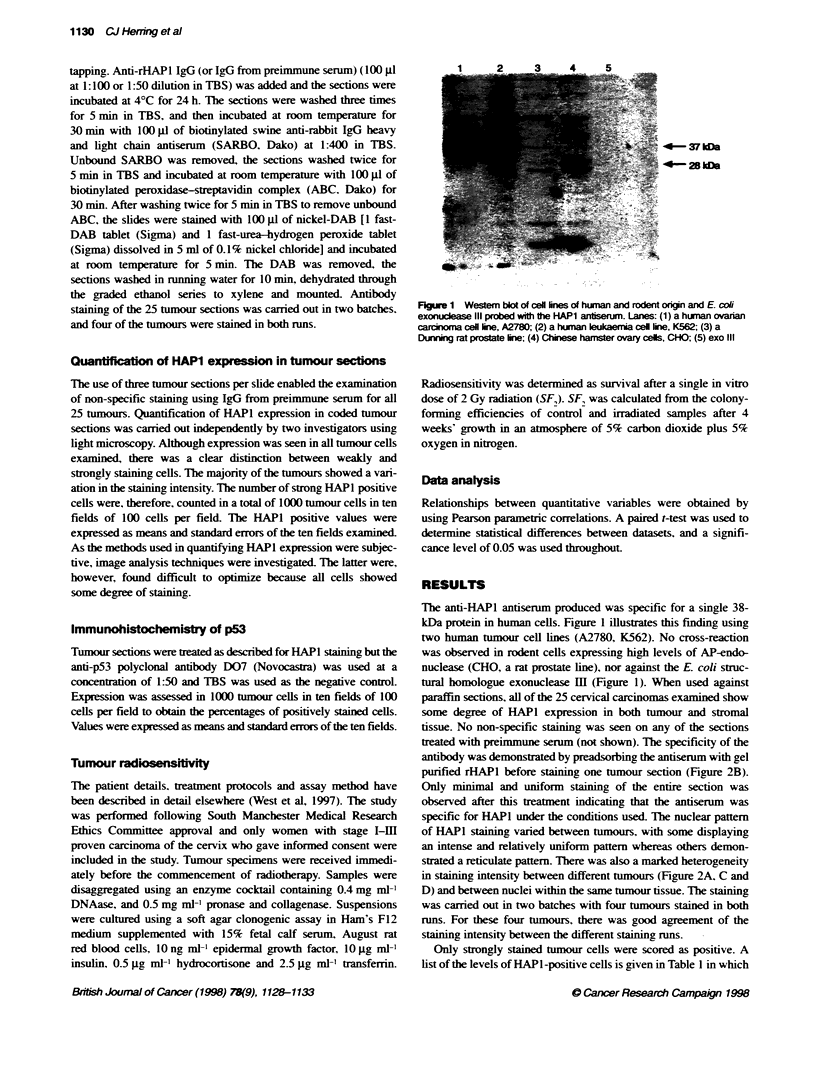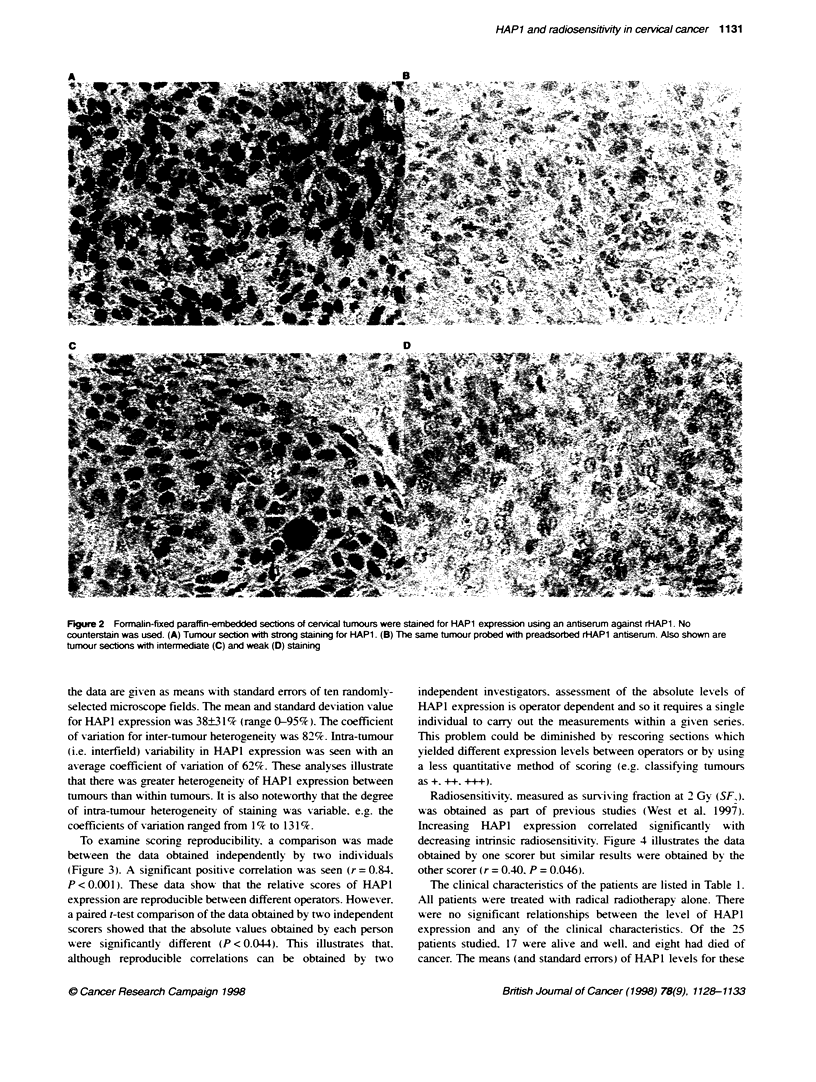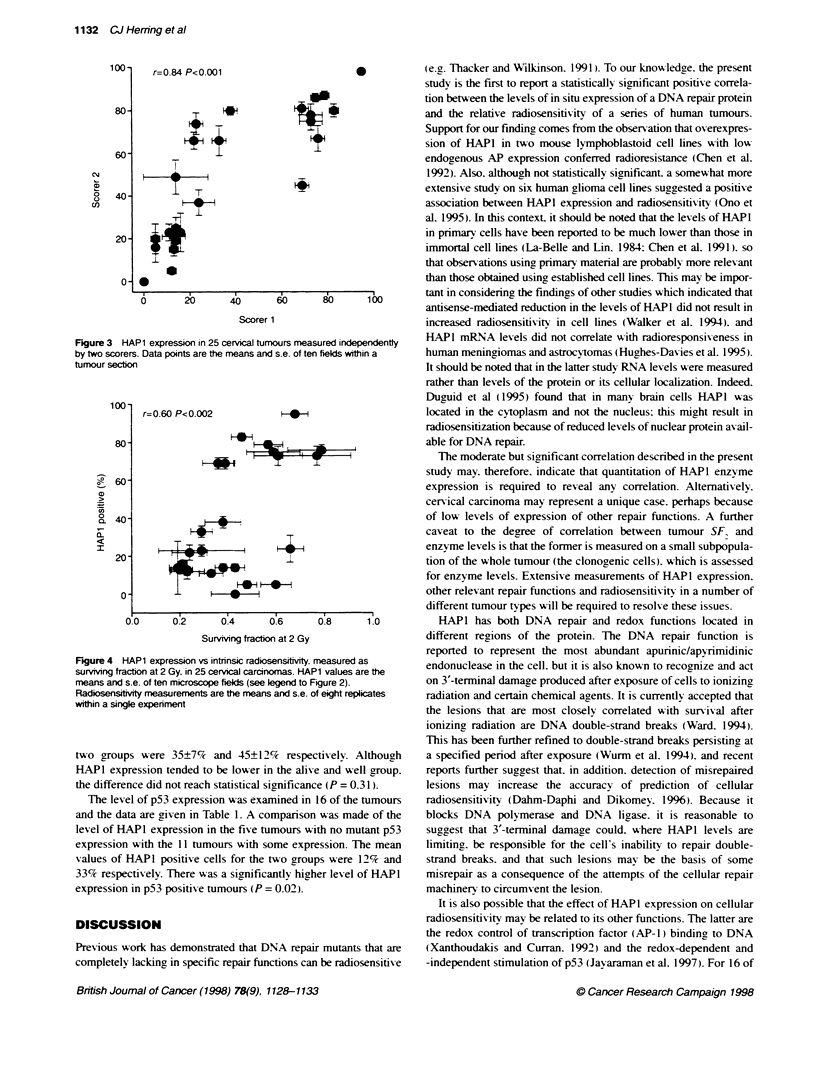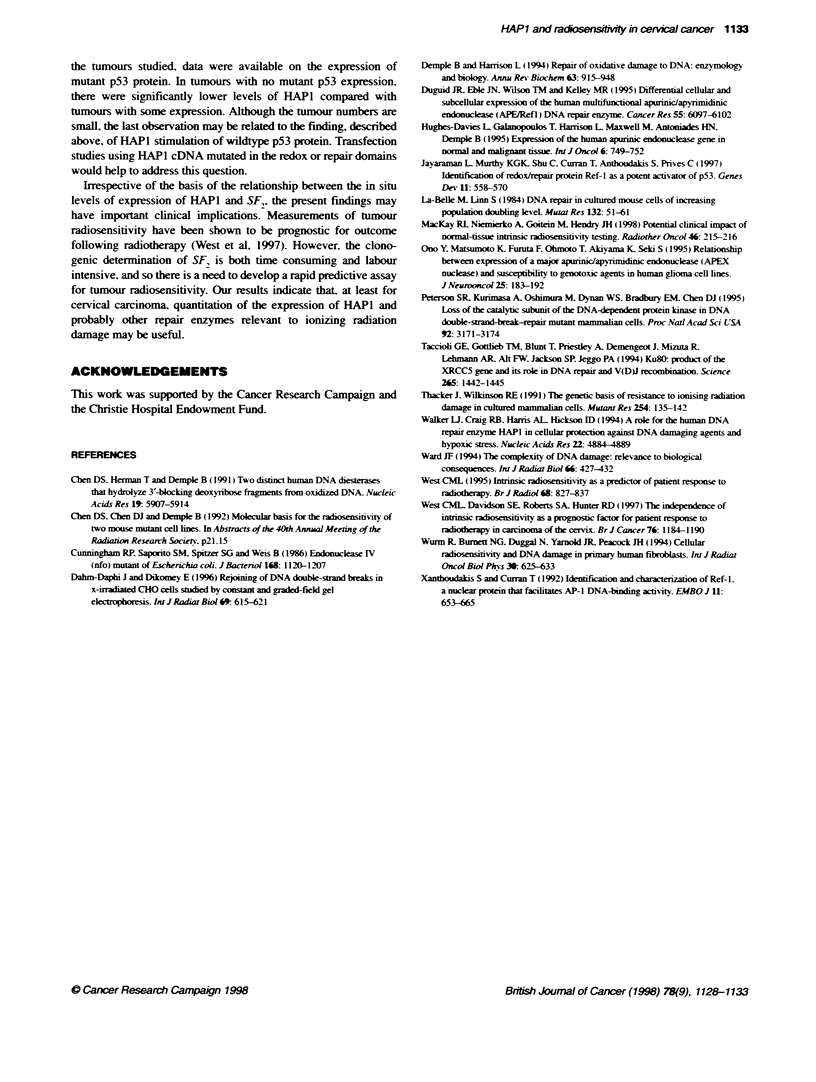# Levels of the DNA repair enzyme human apurinic/apyrimidinic endonuclease (APE1, APEX, Ref-1) are associated with the intrinsic radiosensitivity of cervical cancers.

**DOI:** 10.1038/bjc.1998.641

**Published:** 1998-11

**Authors:** C. J. Herring, C. M. West, D. P. Wilks, S. E. Davidson, R. D. Hunter, P. Berry, G. Forster, J. MacKinnon, J. A. Rafferty, R. H. Elder, J. H. Hendry, G. P. Margison

**Affiliations:** Cancer Research Campaign Section of Genome Damage and Repair, Paterson Institute for Cancer Research, Manchester, UK.

## Abstract

**Images:**


British Journal of Cancer (1998) 78(9), 1128–1133
© 1998 Cancer Research Campaign

### Levels of the DNA repair enzyme human apurinic/apyrimidinic endonuclease (APE1, APEX, Ref-1) are associated with the intrinsic radiosensitivity of cervical cancers

**CJ Herring[1]*, CML West[1], DP Wilks[1], SE Davidson[2], RD Hunter[2], P Berry[1], G Forster[1], J MacKinnon[1], JA Rafferty[1], RH Elder[1], JH Hendry[1] and GP Margison[1]**

[1]Cancer Research Campaign Section of Genome Damage and Repair, Paterson Institute for Cancer Research, and [2]Department of Clinical Oncology, Christie Hospital (NHS) Trust, Wilmslow Road, Manchester M20 4BX, UK

**Summary** A study was made of the relationship between the intrinsic radiosensitivity of human cervical tumours and the expression of the DNA repair enzyme human apurinic/apyrimidinic endonuclease (HAP1). The radiosensitivity of clonogenic cells in tumour biopsies was measured as surviving fraction at 2 Gy ($SF_2$) using a soft agar assay. HAP1 expression levels were determined after staining of formalin-fixed paraffin-embedded tumour sections with a rabbit antiserum raised against recombinant HAP1. Both measurements were obtained on pretreatment biopsy material. All 25 tumours examined showed positive staining for HAP1, but there was heterogeneity in the level of expression both within and between tumours. The average coefficients of variation for intra- and intertumour heterogeneity were 62% and 82% respectively. There was a moderate but significant positive correlation between the levels of HAP1 expression and $SF_2$ ($r = 0.60$, $P = 0.002$). Hence, this study shows that there is some relationship between intrinsic radiosensitivity and expression of a DNA repair enzyme in cervical carcinomas. The results suggest that this type of approach may be useful in the development of rapid predictive tests of tumour radiosensitivity.

**Keywords:** HAP1; predictive assays; $SF_2$; immunohistochemistry; DNA repair

There is considerable interest in increasing the understanding of the factors that determine the radiosensitivity of cells from different individuals (West, 1995). Eventually, these factors may be exploited in the development of rapid assays for predicting the radioresponsiveness of both normal and malignant cells. It has been estimated that the theoretical benefits of radiosensitivity testing could be substantial in terms of improving outcome and reducing morbidity for cancer patients undergoing radiotherapy (MacKay et al, 1998).

The survival of cells following exposure to radiation is governed by complex interactions between a large number of proteins involved in DNA damage recognition, DNA repair and the response of the cells to unrepaired or misrepaired DNA damage. For example, absence of components of the DNA double-strand break recognition complex DNA–PK has been identified as a cause of radiosensitivity in the hamster xrs6 line (lacking the Ku80 protein; Taccioli et al, 1994) and in the *scid* mouse (lacking the p350 protein; Petersen et al, 1995). It is, therefore, reasonable to suggest that variations in the levels of expression of such proteins may result in subtle variations in radiosensitivity.

The major lethal DNA lesion produced by ionizing radiation is considered to be the double-strand break, possibly associated with other types of end-structure damage at the break to constitute 'clustered' damage (Ward, 1994). This may include apurinic/apyrimidinic (AP) sites generated either during initial chemical reactions after irradiation or as a result of the processing of base lesions by components of the base excision repair pathway (see Demple and Harrison, 1994). This latter pathway involves the recognition of damage by a DNA repair glycosylase to produce AP sites, which are subsequently incised by an AP lyase or AP endonuclease. The HAP1 (also known as APE1 and APEX in the mouse) protein is considered to be the major AP endonuclease in human cells and has homology of structure and function to *Escherichia coli* exonuclease III, which has been shown to play an important role in DNA repair and response to ionizing radiation (Cunningham et al, 1986). The HAP1 protein is identical to the redox factor 1 protein (ref1), which has been shown to be involved in the redox regulation of DNA binding of transcription factors such as c-*fos* and c-*jun* (Xanthoudakis and Curran, 1992).

Chen et al (1992) reported that two radiosensitive mouse lymphoblastoid lines showed very low expression levels of APEX as determined by Western analysis and that their radiosensitivity was reduced by transfection with a plasmid encoding HAP1. Furthermore, in data reported by Ono et al (1995), there was a trend towards an association between HAP1 activity and the radiosensitivities of six human glioma cell lines.

The present study was carried out to examine the expression of HAP1 in a series of human cervical carcinomas that had previously been assayed for radiosensitivity using a clonogenic assay (West et al, 1997). The aim was to establish any relationship between the two measurements, and so determine the importance of HAP1 expression in the radiosensitivity of primary cervical carcinoma.

*Received 14 November 1997*
*Revised 24 February 1998*
*Accepted 1 April 1998*

*Correspondence to:* C West

**Current address:* Department of Pathology, University of Cambridge, Tennis Court Road, Cambridge CB2 1QP, UK

**Table 1** HAP1 expression in 25 cervical tumours

| Sample | HAP1 | $SF_2$ | Age | Stage | Histology | p53 | Outcome |
|---|---|---|---|---|---|---|---|
| V41 | 13 ± 2 | 0.20 ± 0.04 | 43 | 3b | Mod. SCC | 30 ± 13 | Alive at 71 months |
| V93 | 14 ± 2 | 0.38 ± 0.03 | 47 | 1b | Well. SCC | 11 ± 2 | Alive at 27 months |
| V109 | 83 ± 2 | 0.46 ± 0.04 | 65 | 1b | Poor. SCC | 14 ± 3 | Alive at 56 months |
| V111 | 69 ± 3 | 0.39 ± 0.04 | 52 | 2a | Poor. SCC | 3 ± 1 | Alive at 64 months |
| V115 | 5 ± 2 | 0.48 ± 0.04 | 40 | 2b | Well. SCC | 0 | Dead at 21 months (PR. METS) |
| V134 | 14 ± 14 | 0.19 ± 0.04 | 44 | 2a | Mod. SCC | 0 | Dead at 32 months (METS) |
| V135 | 23 ± 3 | 0.29 ± 0.18 | 48 | 1b | Mod. SCC | 13 ± 1 | Alive at 54 months |
| V137 | 24 ± 7 | 0.66 ± 0.05 | 44 | 3b | Mod. SCC | 5 ± 1 | Dead at 33 months (METS) |
| V138 | 33 ± 3 | 0.29 ± 0.05 | 57 | 1b | Mod. SCC | 4 ± 1 | Alive at 58 months |
| V144 | 13 ± 5 | 0.23 ± 0.02 | 72 | 1b | Well. SCC | 0 | Alive at 50 months |
| V151 | 16 ± 1 | 0.21 ± 0.02 | 66 | 1b | Mod. SCC | 0 | Dead at 17 months (CR. PR. METS) |
| V154 | 11 ± 1 | 0.33 ± 0.05 | 30 | 2b | Mod. SCC | 0 | Alive at 55 months |
| V157 | 14 ± 2 | 0.43 ± 0.04 | 59 | 2b | Mod. SCC | 35 ± 4 | Alive at 26 months |
| V158 | 0 ± 0 | 0.43 ± 0.10 | 50 | 2b | Mod. SCC | 16 ± 1 | Alive at 34 months |
| V207 | 95 ± 1 | 0.68 ± 0.05 | 49 | 2a | Mod. SCC | 13 ± 1 | Alive at 24 months |
| V209 | 73 ± 5 | 0.77 ± 0.14 | 71 | 1b | Mod. SCC | 13 ± 2 | Alive at 39 months |
| V236 | 79 ± 2 | 0.57 ± 0.06 | 57 | 3b | Mod. SCC | nd | Dead at 32 months (METS) |
| V239 | 12 ± 2 | 0.23 ± 0.07 | 43 | 2b | Poor. adeno | nd | Alive at 35 months |
| V240 | 22 ± 4 | 0.24 ± 0.07 | 71 | 1b | Mod. SCC | nd | Alive at 38 months |
| V243 | 76 ± 3 | 0.79 ± 0.14 | 38 | 3b | Mod. SCC | nd | Dead at 33 months (CR) |
| V269 | 75 ± 2 | 0.59 ± 0.11 | 71 | 3b | SCC | nd | Dead at 22 months (CR. PR. METS) |
| V278 | 5 ± 1 | 0.60 ± 0.06 | 45 | 2b | Well. SCC | nd | Alive at 22 months |
| V296 | 38 ± 3 | 0.38 ± 0.08 | 57 | 2a | Mod. SCC | nd | Alive at 24 months |
| V298 | 69 ± 3 | 0.36 ± 0.07 | 51 | 3b | Mod. SCC | nd | Alive at 11 months |
| V299 | 73 ± 5 | 0.61 ± 0.10 | 37 | 3b | SCC | nd | Dead at 3 months (CR. PR. METS) |

HAP1 values are percentages and are the means and s.e. of ten randomly selected fields. $SF_2$ values are the means and s.e. of 4–12 replicate tubes. Histology is classified as either squamous cell carcinoma (SCC) or adenocarcinoma (adeno) and either well, moderately (mod) or poorly (poor) differentiated. p53 values are percentages and are the means and s.e. of ten randomly selected fields (nd = not done). Patient outcome was classified as central (CR) or peripheral (PR) recurrence, or metastases (METS).

### MATERIALS AND METHODS

### Preparation of HAP1 antiserum

An anti-rHAP1 antiserum was produced by intramuscular injection of 450 µg of rHAP1 in 350 µl of 50% Titermax in phosphate-buffered saline (PBS), split between two sites on each flank of a Half-Lop rabbit. After 37 days, a boost was given of 200 µg of rHAP1 in 200 µl 50% Titermax in PBS, split between two sites. A final boost of another 200 µg of rHAP1 was given as above 57 days later, and blood was collected by cardiac puncture after 13 days. Antisera were screened for reactivity against HAP1 by both Western blotting and antibody capture enzyme-linked immunosorbent assay (ELISA) using rHAP1 polypeptide. Before use in immunohistochemistry, total immunoglobulin G (IgG) was purified from both the preimmune serum and antiserum obtained from the final bleed using a Bio-Rad Econo-Pac serum immunoglobulin G purification kit.

### Antibody specificity

Western blotting was used to demonstrate the specificity of the polyclonal antiserum produced. Cell extracts from two human tumour cell lines (A2780, K562) and two non-human lines (rat Dunning prostate, CHO) were separated by sodium dodecyl sulphate polyacrylamide gel electrophoresis (SDS-PAGE). The separated proteins were then transferred by electroblotting onto Hybond-C Super nitrocellulose membranes (Amersham, UK) using the BioRad Mini Trans-blot apparatus. Transfer was carried out for 1 h at 85 V, in Western transfer buffer (25 mM Tris, 190 mM glycine, 20% methanol). After blotting, the membrane was air dried, wrapped in Saran Wrap and stored at 4°C until processed. The membrane was wetted with PBS containing 0.3% Tween-20 (PBST) and membrane-bound proteins stained with 0.1% black India ink in PBST for approximately 30 min with agitation. Excess ink was removed by washing with PBST for 30 min, and the membrane incubated in TBST blocking buffer (5% non-fat milk in 50 mM Tris-HCl pH 7.5, 150 mM sodium chloride, 0.1% v/v Tween 20) for 1 h at room temperature. The membrane was incubated with the primary antiserum (anti-HAP1 at 1:666 dilution) in blocking buffer for 1.5 h at room temperature. Unbound antibody was removed by washing three times in TBST. The secondary antibody was then applied: goat–anti-rabbit IgG conjugated with horseradish peroxidase (Dako), at a 1:2000 dilution in blocking buffer, for 1 h at room temperature. After three more washes in TBST, bound antibody was detected using a luminol-based chemiluminescence method (ECL, Amersham).

### Immunohistochemistry of HAP1

Formalin-fixed, paraffin-embedded sections (5 µm thick) were dewaxed in xylene for 10 min and rehydrated by passage through a graded ethanol series to tap water. The sections were then treated to unmask antigens by microwaving in 1 l of 10 mM citrate buffer (pH 6.0) for 25 min at 700 W. After cooling for 10 min in the buffer, the sections were cooled to room temperature by the addition of tap water and immersed in Tris-buffered saline (TBS). Endogenous peroxidases were blocked with 3% hydrogen peroxide in TBS for 20 min and the sections washed twice for 5 min in TBS. The sections were then blocked further using 10% normal swine serum (Dako) in TBS and the serum removed by

© Cancer Research Campaign 1998

tapping. Anti-rHAP1 IgG (or IgG from preimmune serum) (100 µl at 1:100 or 1:50 dilution in TBS) was added and the sections were incubated at 4°C for 24 h. The sections were washed three times for 5 min in TBS, and then incubated at room temperature for 30 min with 100 µl of biotinylated swine anti-rabbit IgG heavy and light chain antiserum (SARBO, Dako) at 1:400 in TBS. Unbound SARBO was removed, the sections washed twice for 5 min in TBS and incubated at room temperature with 100 µl of biotinylated peroxidase–streptavidin complex (ABC, Dako) for 30 min. After washing twice for 5 min in TBS to remove unbound ABC, the slides were stained with 100 µl of nickel-DAB [1 fast-DAB tablet (Sigma) and 1 fast-urea–hydrogen peroxide tablet (Sigma) dissolved in 5 ml of 0.1% nickel chloride] and incubated at room temperature for 5 min. The DAB was removed, the sections washed in running water for 10 min, dehydrated through the graded ethanol series to xylene and mounted. Antibody staining of the 25 tumour sections was carried out in two batches, and four of the tumours were stained in both runs.

**Figure 1** Western blot of cell lines of human and rodent origin and *E. coli* exonuclease III probed with the HAP1 antiserum. Lanes: (1) a human ovarian carcinoma cell line, A2780; (2) a human leukaemia cell line, K562; (3) a Dunning rat prostate line; (4) Chinese hamster ovary cells, CHO; (5) exo III

### Quantification of HAP1 expression in tumour sections

The use of three tumour sections per slide enabled the examination of non-specific staining using IgG from preimmune serum for all 25 tumours. Quantification of HAP1 expression in coded tumour sections was carried out independently by two investigators using light microscopy. Although expression was seen in all tumour cells examined, there was a clear distinction between weakly and strongly staining cells. The majority of the tumours showed a variation in the staining intensity. The number of strong HAP1 positive cells were, therefore, counted in a total of 1000 tumour cells in ten fields of 100 cells per field. The HAP1 positive values were expressed as means and standard errors of the ten fields examined. As the methods used in quantifying HAP1 expression were subjective, image analysis techniques were investigated. The latter were, however, found difficult to optimize because all cells showed some degree of staining.

### Immunohistochemistry of p53

Tumour sections were treated as described for HAP1 staining but the anti-p53 polyclonal antibody DO7 (Novocastra) was used at a concentration of 1:50 and TBS was used as the negative control. Expression was assessed in 1000 tumour cells in ten fields of 100 cells per field to obtain the percentages of positively stained cells. Values were expressed as means and standard errors of the ten fields.

### Tumour radiosensitivity

The patient details, treatment protocols and assay method have been described in detail elsewhere (West et al, 1997). The study was performed following South Manchester Medical Research Ethics Committee approval and only women with stage I–III proven carcinoma of the cervix who gave informed consent were included in the study. Tumour specimens were received immediately before the commencement of radiotherapy. Samples were disaggregated using an enzyme cocktail containing 0.4 mg $ml^{-1}$ DNAase, and 0.5 mg $ml^{-1}$ pronase and collagenase. Suspensions were cultured using a soft agar clonogenic assay in Ham's F12 medium supplemented with 15% fetal calf serum, August rat red blood cells, 10 ng $ml^{-1}$ epidermal growth factor, 10 µg $ml^{-1}$ insulin, 0.5 µg $ml^{-1}$ hydrocortisone and 2.5 µg $ml^{-1}$ transferrin. Radiosensitivity was determined as survival after a single in vitro dose of 2 Gy radiation ($SF_2$). $SF_2$ was calculated from the colony-forming efficiencies of control and irradiated samples after 4 weeks' growth in an atmosphere of 5% carbon dioxide plus 5% oxygen in nitrogen.

### Data analysis

Relationships between quantitative variables were obtained by using Pearson parametric correlations. A paired *t*-test was used to determine statistical differences between datasets, and a significance level of 0.05 was used throughout.

### RESULTS

The anti-HAP1 antiserum produced was specific for a single 38-kDa protein in human cells. Figure 1 illustrates this finding using two human tumour cell lines (A2780, K562). No cross-reaction was observed in rodent cells expressing high levels of AP-endonuclease (CHO, a rat prostate line), nor against the *E. coli* structural homologue exonuclease III (Figure 1). When used against paraffin sections, all of the 25 cervical carcinomas examined show some degree of HAP1 expression in both tumour and stromal tissue. No non-specific staining was seen on any of the sections treated with preimmune serum (not shown). The specificity of the antibody was demonstrated by preadsorbing the antiserum with gel purified rHAP1 before staining one tumour section (Figure 2B). Only minimal and uniform staining of the entire section was observed after this treatment indicating that the antiserum was specific for HAP1 under the conditions used. The nuclear pattern of HAP1 staining varied between tumours, with some displaying an intense and relatively uniform pattern whereas others demonstrated a reticulate pattern. There was also a marked heterogeneity in staining intensity between different tumours (Figure 2A, C and D) and between nuclei within the same tumour tissue. The staining was carried out in two batches with four tumours stained in both runs. For these four tumours, there was good agreement of the staining intensity between the different staining runs.

Only strongly stained tumour cells were scored as positive. A list of the levels of HAP1-positive cells is given in Table 1 in which

© Cancer Research Campaign 1998

**Figure 2** Formalin-fixed paraffin-embedded sections of cervical tumours were stained for HAP1 expression using an antiserum against rHAP1. No counterstain was used. (**A**) Tumour section with strong staining for HAP1. (**B**) The same tumour probed with preadsorbed rHAP1 antiserum. Also shown are tumour sections with intermediate (**C**) and weak (**D**) staining the data are given as means with standard errors of ten randomly-selected microscope fields. The mean and standard deviation value for HAP1 expression was 38±31% (range 0–95%). The coefficient of variation for inter-tumour heterogeneity was 82%. Intra-tumour (i.e. interfield) variability in HAP1 expression was seen with an average coefficient of variation of 62%. These analyses illustrate that there was greater heterogeneity of HAP1 expression between tumours than within tumours. It is also noteworthy that the degree of intra-tumour heterogeneity of staining was variable, e.g. the coefficients of variation ranged from 1% to 131%.

To examine scoring reproducibility, a comparison was made between the data obtained independently by two individuals (Figure 3). A significant positive correlation was seen ($r = 0.84$, $P < 0.001$). These data show that the relative scores of HAP1 expression are reproducible between different operators. However, a paired *t*-test comparison of the data obtained by two independent scorers showed that the absolute values obtained by each person were significantly different ($P < 0.044$). This illustrates that, although reproducible correlations can be obtained by two independent investigators, assessment of the absolute levels of HAP1 expression is operator dependent and so it requires a single individual to carry out the measurements within a given series. This problem could be diminished by rescoring sections which yielded different expression levels between operators or by using a less quantitative method of scoring (e.g. classifying tumours as +, ++, +++).

Radiosensitivity, measured as surviving fraction at 2 Gy ($SF_2$), was obtained as part of previous studies (West et al, 1997). Increasing HAP1 expression correlated significantly with decreasing intrinsic radiosensitivity. Figure 4 illustrates the data obtained by one scorer but similar results were obtained by the other scorer ($r = 0.40$, $P = 0.046$).

The clinical characteristics of the patients are listed in Table 1. All patients were treated with radical radiotherapy alone. There were no significant relationships between the level of HAP1 expression and any of the clinical characteristics. Of the 25 patients studied, 17 were alive and well, and eight had died of cancer. The means (and standard errors) of HAP1 levels for these

© Cancer Research Campaign 1998

**Figure 3** HAP1 expression in 25 cervical tumours measured independently by two scorers. Data points are the means and s.e. of ten fields within a tumour section

**Figure 4** HAP1 expression vs intrinsic radiosensitivity, measured as surviving fraction at 2 Gy, in 25 cervical carcinomas. HAP1 values are the means and s.e. of ten microscope fields (see legend to Figure 2). Radiosensitivity measurements are the means and s.e. of eight replicates within a single experiment two groups were 35±7% and 45±12% respectively. Although HAP1 expression tended to be lower in the alive and well group, the difference did not reach statistical significance ($P = 0.31$).

The level of p53 expression was examined in 16 of the tumours and the data are given in Table 1. A comparison was made of the level of HAP1 expression in the five tumours with no mutant p53 expression with the 11 tumours with some expression. The mean values of HAP1 positive cells for the two groups were 12% and 33% respectively. There was a significantly higher level of HAP1 expression in p53 positive tumours ($P = 0.02$).

### DISCUSSION

Previous work has demonstrated that DNA repair mutants that are completely lacking in specific repair functions can be radiosensitive (e.g. Thacker and Wilkinson, 1991). To our knowledge, the present study is the first to report a statistically significant positive correlation between the levels of in situ expression of a DNA repair protein and the relative radiosensitivity of a series of human tumours. Support for our finding comes from the observation that overexpression of HAP1 in two mouse lymphoblastoid cell lines with low endogenous AP expression conferred radioresistance (Chen et al, 1992). Also, although not statistically significant, a somewhat more extensive study on six human glioma cell lines suggested a positive association between HAP1 expression and radiosensitivity (Ono et al, 1995). In this context, it should be noted that the levels of HAP1 in primary cells have been reported to be much lower than those in immortal cell lines (La-Belle and Lin, 1984; Chen et al, 1991), so that observations using primary material are probably more relevant than those obtained using established cell lines. This may be important in considering the findings of other studies which indicated that antisense-mediated reduction in the levels of HAP1 did not result in increased radiosensitivity in cell lines (Walker et al, 1994), and HAP1 mRNA levels did not correlate with radioresponsiveness in human meningiomas and astrocytomas (Hughes-Davies et al, 1995). It should be noted that in the latter study RNA levels were measured rather than levels of the protein or its cellular localization. Indeed, Duguid et al (1995) found that in many brain cells HAP1 was located in the cytoplasm and not the nucleus; this might result in radiosensitization because of reduced levels of nuclear protein available for DNA repair.

The moderate but significant correlation described in the present study may, therefore, indicate that quantitation of HAP1 enzyme expression is required to reveal any correlation. Alternatively, cervical carcinoma may represent a unique case, perhaps because of low levels of expression of other repair functions. A further caveat to the degree of correlation between tumour $SF_2$ and enzyme levels is that the former is measured on a small subpopulation of the whole tumour (the clonogenic cells), which is assessed for enzyme levels. Extensive measurements of HAP1 expression, other relevant repair functions and radiosensitivity in a number of different tumour types will be required to resolve these issues.

HAP1 has both DNA repair and redox functions located in different regions of the protein. The DNA repair function is reported to represent the most abundant apurinic/apyrimidinic endonuclease in the cell, but it is also known to recognize and act on 3′-terminal damage produced after exposure of cells to ionizing radiation and certain chemical agents. It is currently accepted that the lesions that are most closely correlated with survival after ionizing radiation are DNA double-strand breaks (Ward, 1994). This has been further refined to double-strand breaks persisting at a specified period after exposure (Wurm et al, 1994), and recent reports further suggest that, in addition, detection of misrepaired lesions may increase the accuracy of prediction of cellular radiosensitivity (Dahm-Daphi and Dikomey, 1996). Because it blocks DNA polymerase and DNA ligase, it is reasonable to suggest that 3′-terminal damage could, where HAP1 levels are limiting, be responsible for the cell's inability to repair double-strand breaks, and that such lesions may be the basis of some misrepair as a consequence of the attempts of the cellular repair machinery to circumvent the lesion.

It is also possible that the effect of HAP1 expression on cellular radiosensitivity may be related to its other functions. The latter are the redox control of transcription factor (AP-1) binding to DNA (Xanthoudakis and Curran, 1992) and the redox-dependent and -independent stimulation of p53 (Jayaraman et al, 1997). For 16 of

© Cancer Research Campaign 1998

the tumours studied, data were available on the expression of mutant p53 protein. In tumours with no mutant p53 expression, there were significantly lower levels of HAP1 compared with tumours with some expression. Although the tumour numbers are small, the last observation may be related to the finding, described above, of HAP1 stimulation of wildtype p53 protein. Transfection studies using HAP1 cDNA mutated in the redox or repair domains would help to address this question.

Irrespective of the basis of the relationship between the in situ levels of expression of HAP1 and $SF_2$, the present findings may have important clinical implications. Measurements of tumour radiosensitivity have been shown to be prognostic for outcome following radiotherapy (West et al, 1997). However, the clonogenic determination of $SF_2$ is both time consuming and labour intensive, and so there is a need to develop a rapid predictive assay for tumour radiosensitivity. Our results indicate that, at least for cervical carcinoma, quantitation of the expression of HAP1 and probably other repair enzymes relevant to ionizing radiation damage may be useful.

### ACKNOWLEDGEMENTS

This work was supported by the Cancer Research Campaign and the Christie Hospital Endowment Fund.

### REFERENCES

Chen DS, Herman T and Demple B (1991) Two distinct human DNA diesterases that hydrolyze 3'-blocking deoxyribose fragments from oxidized DNA. *Nucleic Acids Res* **19**: 5907–5914

Chen DS, Chen DJ and Demple B (1992) Molecular basis for the radiosensitivity of two mouse mutant cell lines. In *Abstracts of the 40th Annual Meeting of the Radiation Research Society*, p21.15

Cunningham RP, Saporito SM, Spitzer SG and Weis B (1986) Endonuclease IV (nfo) mutant of *Escherichia coli*. *J Bacteriol* **168**: 1120–1207

Dahm-Daphi J and Dikomey E (1996) Rejoining of DNA double-strand breaks in x-irradiated CHO cells studied by constant and graded-field gel electrophoresis. *Int J Radiat Biol* **69**: 615–621

Demple B and Harrison L (1994) Repair of oxidative damage to DNA: enzymology and biology. *Annu Rev Biochem* **63**: 915–948

Duguid JR, Eble JN, Wilson TM and Kelley MR (1995) Differential cellular and subcellular expression of the human multifunctional apurinic/apyrimidinic endonuclease (APE/Ref1) DNA repair enzyme. *Cancer Res* **55**: 6097–6102

Hughes-Davies L, Galanopoulos T, Harrison L, Maxwell M, Antoniades HN, Demple B (1995) Expression of the human apurinic endonuclease gene in normal and malignant tissue. *Int J Oncol* **6**: 749–752

Jayaraman L, Murthy KGK, Shu C, Curran T, Anthoudakis S, Prives C (1997) Identification of redox/repair protein Ref-1 as a potent activator of p53. *Genes Dev* **11**: 558–570

La-Belle M, Linn S (1984) DNA repair in cultured mouse cells of increasing population doubling level. *Mutat Res* **132**: 51–61

MacKay RI, Niemierko A, Goitein M, Hendry JH (1998) Potential clinical impact of normal-tissue intrinsic radiosensitivity testing. *Radiother Oncol* **46**: 215–216

Ono Y, Matsumoto K, Furuta F, Ohmoto T, Akiyama K, Seki S (1995) Relationship between expression of a major apurinic/apyrimidinic endonuclease (APEX nuclease) and susceptibility to genotoxic agents in human glioma cell lines. *J Neurooncol* **25**: 183–192

Peterson SR, Kurimasa A, Oshimura M, Dynan WS, Bradbury EM, Chen DJ (1995) Loss of the catalytic subunit of the DNA-dependent protein kinase in DNA double-strand-break–repair mutant mammalian cells. *Proc Natl Acad Sci USA* **92**: 3171–3174

Taccioli GE, Gottlieb TM, Blunt T, Priestley A, Demengeot J, Mizuta R, Lehmann AR, Alt FW, Jackson SP, Jeggo PA (1994) Ku80: product of the XRCC5 gene and its role in DNA repair and V(D)J recombination. *Science* **265**: 1442–1445

Thacker J, Wilkinson RE (1991) The genetic basis of resistance to ionising radiation damage in cultured mammalian cells. *Mutant Res* **254**: 135–142

Walker LJ, Craig RB, Harris AL, Hickson ID (1994) A role for the human DNA repair enzyme HAP1 in cellular protection against DNA damaging agents and hypoxic stress. *Nucleic Acids Res* **22**: 4884–4889

Ward JF (1994) The complexity of DNA damage: relevance to biological consequences. *Int J Radiat Biol* **66**: 427–432

West CML (1995) Intrinsic radiosensitivity as a predictor of patient response to radiotherapy. *Br J Radiol* **68**: 827–837

West CML, Davidson SE, Roberts SA, Hunter RD (1997) The independence of intrinsic radiosensitivity as a prognostic factor for patient response to radiotherapy in carcinoma of the cervix. *Br J Cancer* **76**: 1184–1190

Wurm R, Burnett NG, Duggal N, Yarnold JR, Peacock JH (1994) Cellular radiosensitivity and DNA damage in primary human fibroblasts. *Int J Radiat Oncol Biol Phys* **30**: 625–633

Xanthoudakis S and Curran T (1992) Identification and characterization of Ref-1, a nuclear protein that facilitates AP-1 DNA-binding activity. *EMBO J* **11**: 653–665

© Cancer Research Campaign 1998